# Initiating second-line antidiabetic medication among older adults with type 2 diabetes on Metformin

**DOI:** 10.1186/s12877-022-02792-3

**Published:** 2022-02-03

**Authors:** Kristen DeCarlo, Amisha Wallia, Raymond H. Kang, Andrew Cooper, Manisha Cherupally, Sterling A. Harris, Cassandra Aikman, David T. Liss, Ronald T. Ackermann, Matthew J. O’Brien

**Affiliations:** 1 Division of Endocrinology, Diabetes and Metabolism, Department of Medicine, University of Illinois at Chicago, 835 Wolcott St. Suite 625E M/C 640, IL 60612 Chicago, USA; 2 Division of Endocrinology, Metabolism and Molecular Medicine, Department of Medicine, Northwestern University Feinberg School of Medicine, 300 E. Superior St. #15-703, IL 60611 Chicago, USA; 3 Institute of Public Health and Medicine, Northwestern University Feinberg School of Medicine, 420 E. Superior St. 9th Floor, IL 60611 Chicago, USA; 4 Division of General Internal Medicine and Geriatrics, Department of Medicine, Northwestern University Feinberg School of Medicine, 750 N. Lake Shore Dr., 10th floor, IL Chicago, USA

**Keywords:** Type 2 diabetes, Older adults, Antidiabetic medications, Sulfonylureas, Insulin

## Abstract

**Background:**

Antidiabetic medications (ADM), especially sulfonylureas (SFU) and basal insulin (BI), are associated with increased risk of hypoglycemia, which is especially concerning among older adults in poor health. The objective of this study was to investigate prescribing patterns of ADM in older adults according to their health status.

**Methods:**

This case control study analyzed administrative claims between 2013 and 2017 from a large national payer. The study population was derived from a nationwide database of 84,720 U.S. adults aged ≥65, who were enrolled in Medicare Advantage health insurance plans. Participants had type 2 diabetes on metformin monotherapy, and started a second-line ADM during the study period. The exposure was a binary variable for health status, with poor health defined by end-stage medical conditions, dementia, or residence in a long-term nursing facility. The outcome was a variable identifying which second-line ADM class was started, categorized as SFU, BI, or other (i.e. all other ADM classes combined).

**Results:**

Over half of participants (54%) received SFU as initial second-line ADM, 14% received BI, and 32% received another ADM. In multivariable models, the odds of filling SFU or BI was higher for participants in poor health than those in good or intermediate health [OR 1.13 (95% CI 1.05-1.21) and OR 2.34 (95% CI 2.14-2.55), respectively]. SFU and BI were also more commonly filled by older adults with poor glycemic control.

**Conclusions:**

Despite clinical consensus to use caution prescribing SFU and BI among older adults in poor health, these medications remain frequently used in this particularly vulnerable population.

## Background

The care of older adults with type 2 diabetes presents a growing public health and safety challenge. Diabetes currently affects 27% of U.S. adults aged 65 years or older, with greater prevalence observed as age increases [1]. Prior research in this population has reported increased risk of diabetes complications, geriatric syndromes, adverse medication events, and associated morbidity and mortality [2]. In light of these concerning data, diabetes care guidelines for older adults recommend developing individualized treatment plans that aim to mitigate these poor outcomes [3–7].

Minimizing hypoglycemia represents a cornerstone of diabetes care in the older population. Older adults are more likely to experience hypoglycemia due to their high rates of polypharmacy, comorbidity, kidney disease, cognitive impairment, and physical frailty, among other risk factors [8, 9]. Hypoglycemia can also result directly from antidiabetic medications (ADM), especially sulfonylureas (SFU) and basal insulin (BI). A recent analysis of nationwide administrative claims data found the highest rates of hypoglycemia associated with these two ADM classes [10]. Despite clinical consensus regarding relaxed glycemic targets among older adults with poor health, several previous studies demonstrate overuse of SFU and BI in this particularly vulnerable population [11–17]. Current diabetes management guidelines address this important area of patient safety by recommending a framework that considers older adults’ health status when prescribing these high-risk ADM [3, 4, 6, 18]. Specifically, the American Diabetes Association (ADA) and American Geriatrics Society (AGS) consensus report, as well as the recent Endocrine Society Guidelines, suggest higher glycemic targets when using SFU and BI in older adults with poor health, which is defined by the presence of end-stage medical conditions, moderate to severe dementia, functional impairment, or residence in a long-term nursing facility [3, 4, 6, 18].

In this study, we aimed to investigate the magnitude of an existing gap between consensus recommendations about diabetes management in older adults and real-world ADM prescribing patterns in this population. Specifically, we examined fills of SFU, BI, and other ADM classes according to health status in a large national population of adults aged 65 years or older who were enrolled in Medicare Advantage insurance plans.

### Methods

#### Study Design

We conducted a case control study using administrative claims data from a large U.S. payer to compare the health status of patients who filled SFU or BI vs. all other ADM alternatives (N=84,720). To minimize the potential for differences in diabetes duration or severity that may influence ADM prescribing decisions, we restricted our analysis to patients who had only taken metformin before starting one of the follow index medication classes: SFU, BI, dipeptidyl peptidase 4 (DPP-4) inhibitors, glucagon-like peptide-1 (GLP-1) agonists, sodium-glucose cotransporter 2 (SGLT-2) inhibitors, or thiazolidinediones (TZD).

### Data and Setting

The data sources were patients’ health plan enrollment files, laboratory claims, pharmacy claims, and medical claims collected by a large health payer between January 1, 2013 and December 31, 2017. Medical diagnoses were coded according to the International Classification of Diseases. Pharmacy claims for individual second-line ADM were grouped into one of the six classes. Hemoglobin A_1c_ (HbA_1c_) values were available for a subset of patients based on their lab vendor. Race/ethnicity is not routinely collected in health plan administrative data sources, but was imputed by the data vendor from regional and other individual characteristics. The data sources studied here were described in-depth previously [19]. We followed the Strengthening the Reporting of Observational Studies in Epidemiology (STROBE) guidelines [20].

### Participants

The study population was derived from a nationwide database of U.S. adults aged ≥65 years, who were enrolled in Medicare Advantage health insurance plans. We included those with: (1) diagnosed type 2 diabetes; (2) a first pharmacy-dispensing event for second-line ADM, defined as the index date; (3) at least 6 months of continuous enrollment before the index date; (4) at least 1 prior fill of metformin during the 6 months before the index date, with no other ADM fills during that time; and (5) at least 1 fill of the index ADM. We considered patients to have type 2 diabetes if they had at least one medical claim associated with a corresponding diagnosis code occurring on or before the index date. We excluded patients with evidence of type 1 diabetes or secondary diabetes prior to the index date, as well as those who filled more than one ADM class on the index date. Detailed definitions of the eligibility criteria, as well as the exposure, are presented in Tables 1 and 2 respectively, with the corresponding diagnosis codes from the International Classification of Diseases [1, 21]. Medications comprising the ADM classes studied here are presented in Table 3.

**Table 1:** Definitions for Diabetes-Related Eligibility Criteria

Condition	ICD-9 Codes^a^	ICD-10 Codes^a^
Inclusion Criteria^b^		
Type 2 diabetes	250.00, 250.02, 250.10, 250.12, 250.20, 250.22, 250.30, 250.32, 250.40, 250.42, 250.50, 250.52, 250.60, 250.62, 250.70, 250.72, 250.80, 250.82, 250.90, 250.92	E11.0x, E11.1x, E11.2x, E11.3xxx, E11.4x, E11.5x, E11.6xx, E11.8, E11.9
Exclusion Criteria		
Type 1 diabetes	250.11, 250.13, 250.21, 250.23, 250.31, 250.33, 250.41, 250.43, 250.51, 250.53, 250.61, 250.63, 250.71, 250.73, 250.81, 250.83, 250.91, 250.93	E10.1x, E10.2x, E10.3xxx E10.4x, E10.5x, E10.6xx, E10.8, E10.9
Secondary diabetes	249.0x, 249.1x, 249.2x, 249.3x, 249.4x, 249.5x, 249.6x, 249.7x, 249.8x, 249.9x	E08.0x, E08.1x, E08.2x, E08.3xxx, E08.4x, E08.5x, E08.6xx, E08.8, E08.9
Started >1 antidiabetic medication on index date^c^	N/A	N/A

^a^ Diagnosis codes were derived from the International Classification of Diseases, Ninth and Tenth Revisions

^b^ All eligibility criteria, in addition to type 2 diabetes, are described in the manuscript under Participants

^c^Medication fills were assessed using pharmacy claims

**Table 2:** Definitions for Conditions That Comprise Poor Health Status

Condition	ICD-9 Codes	ICD-10 Codes	Billing Codes
End-Stage Medical Conditions			
Metastatic cancer	196.x - 199.x	C77.x - C80.x	N/A
Oxygen-dependent lung disease	V46.2	Z99.81	N/A
Chronic kidney disease, stages IV-V	585.4, 585.5	N18.4, N18.5	N/A
End-stage renal disease	585.6, V45.11	N18.6, Z99.2	N/A
Heart failure^a^	398.91, 402.01, 402.11, 402.91, 404.01, 404.03, 404.11, 404.13, 404.91, 404.93, 425.4, 425.5, 425.7, 425.8, 425.9, 428.x, 37.66, 37.51, 37.52, V42.1, 996.83, V43.21	I09.9, I11.0, I13.0, I13.2, I25.5, I42.0, I42.5, I42.6, I42.7, I42.8, 142.9, I43.x, I50.x, Z95.811, T86.2x, Z48.21, Z94.1, 02HA0QZ, 02YA0Z0, 02YA0Z1,	N/A
Dementia	331.0, 331.01, 331.11, 331.19, 331.2, 331.7, 331.82 290.0, 290.10, 290.11, 290.12, 290.13, 290.20, 290.21, 290.3, 290.10, 290.41, 290.42, 290.43, 291.2, 294.0, 294.10, 294.11, 294.20, 294.21, 294.8, 797	F01.50, F01.51, F02.80, F02.81, F03.90, F03.91, F04, G13.8, F05, F06.1, F06.8, F10.27, G30.0, G30.1, G30.8, G30.9, G31.1, G31.2, G31.01, G31.09, G31.83, G94, R41.81, R54	N/A
Long-term nursing facility residence^b^			99301-99303, 99311-99313, 99,315, 99,316, 99,379, 99,380, G0066

^a^ Heart failure was defined as any code listed above + ≥1 hospitalization or ED visit for heart failure within 6 months of the index date

^b^ Billing codes for residence in a long-term nursing facility include CPT codes and nursing facility place of service codes

**Table 3:** Individual Medications within Each Antidiabetic Medication Class

Antidiabetic Medication Class	Generic Name	Brand Names
Medications of Interest		
SFU	glipizide	Glucotrol
	glyburide	Diabeta, Micronase, Glynase
	glicazide	Diamicron
	glimepiride	Amaryl
	repaglinide	Prandin
	nateglinide	Starlix
Basal insulin	insulin glargine	Lantus, Toujeo, Basaglar
	insulin detemir	Levemir
	insulin degludec	Tresiba
	insulin NPH	Humulin, Novolin
Other Medications		
DPP-4	sitagliptin	Januvia
	saxagliptin	Onglyza
	alogliptin	Nesina
	linagliptin	Tradjenta
	vildagliptin	Gliptus, Galvus
GLP-1	exenatide	Bydureon, Bydureon BCise, Byetta
	lixisenatide	Adlyxin
	liraglutide	Saxenda, Victoza
	albiglutide	Tanzeum
	dulaglutide	Trulicity
	semaglutide	Ozempic
SGLT-2	empagliflozin	Jardiance
	canagliflozin	Invokana
	dapagliflozin	Farxiga
TZD	rosiglitazone	Avandia
	pioglitazone	Actos

*DPP-4 *dipeptidyl peptidase-4 inhibitors; *GLP-1 *glucagon-like peptide 1 receptor agonists; *NPH *neutral protamine hagedorn; *SGLT-2 *sodium-glucose cotransporter 2 inhibitors; *TZD* thiazolidinediones; *SFU* sulfonylureas and meglitanides

### Exposure and Outcomes

The exposure was a binary variable for health status, adapted from consensus guidelines focused on diabetes management in older adults [3, 4, 6]. Poor health was defined by the presence of at least one medical claim with a diagnosis code for end-stage medical conditions, dementia, or residence in a long-term nursing facility. Patients without evidence of any of these criteria were assumed to be in good or intermediate health. Impairment in activities of daily living could not be assessed using administrative claims data, and was therefore not included in our primary exposure variable. End-stage conditions comprising the guideline definition of poor health was determined by the presence of medical claims associated with diagnosis codes for metastatic cancer, oxygen-dependent lung disease, severe chronic kidney disease (i.e. stages 4, 5, end-stage kidney disease, or dialysis status), and congestive heart failure (Table 3) prior to the index date [3, 4, 6]. The outcome was a categorical variable identifying which second-line ADM class was started on the index date, defined by the first fill of the new ADM. Individual index ADM pharmacy claims were assigned to one of the following categories: SFU, BI, or other (i.e. DPP-4, GLP-1, SGLT-2, or TZD).

### Covariates

All covariates were assessed within the 6 months preceding the index date. Demographic data included patients’ age, sex, and race/ethnicity. Laboratory values are not routinely available in health plan administrative data sources unless submitted by the laboratory vendor as part of their contract with the health payer. For these data, we were fortunate to have access to a laboratory result for 35% of submitted laboratory claims nationally. The most recent HbA_1c_ result was categorized: <8.0%, 8.0-10.0%, >10.0%, and a separate category for those without available values. We used diagnostic codes from inpatient and ambulatory medical claims to examine prevalent micro- and macrovascular complications prior to the index date. These conditions were grouped according to the Diabetes Complications Severity Index (DCSI) [22], and categorized into the following DCSI scores: 0, 1, 2, and ≥3. Cardiovascular codes in the DCSI were excluded because they comprise end-stage conditions that were used to define the primary exposure, thereby reducing the total DCSI score by a maximum of 2 points. Characteristics of prescribers (i.e. family physician, general/internal medicine physician, endocrinologist, nurse practitioner, physician assistant, missing or other) and health coverage (i.e. plan type) were also analyzed as covariates, as well as the year in which the index ADM was prescribed.

### Statistical Analysis

Summary statistics characterized the study population by the class of the index ADM (Table 4), using Chi-square tests to examine associations between baseline covariates and ADM class. Table 5 presents the association of the exposure by index ADM class. The exposure was examined as a dichotomous variable for poor health vs. good or intermediate health, in addition to the individual criteria comprising the definition of poor health (i.e. end-stage comorbidities, dementia, and residence in a long-term nursing facility). Logistic regression was used to model the multivariable association between health status and index ADM class. Two separate multivariable models were constructed, namely: (1) SFU vs. all other ADM alternatives, excluding BI; and (2) BI vs. all other ADM alternatives, excluding SFU.

We conducted a sensitivity analysis excluding the imputed race/ethnicity variable from logistic regression models. We also conducted a sensitivity analysis among only those patients with available HbA_1c_ values prior to the index date. All covariates in this sensitivity analysis were modeled in an identical manner as the primary analysis. All analyses were conducted using [SAS version 9.4, Cary, NC]. The Northwestern University IRB deemed this study to be non-human subjects research, and therefore written informed consent of participants was not required.

**Table 4:** Patient Characteristics by Initial Second-Line Antidiabetic Medication Class

Characteristic^**a**^	Total	SFU	BI	Other^**b**^
	N (%)	N (%)	N (%)	N (%)
Total	84,720 (100)	45,734 (54)	12,165 (14)	26,821 (32)
Sex				
Female	43,236 (51)	22,528 (49)	6,683 (55)	14,025 (52)
Male	41,484 (49)	23,206 (51)	5,482 (45)	12,796 (48)
Age, years				
65-67	22,425 (26)	11,540 (25)	3,604 (30)	7,281 (27)
68-70	13,145 (16)	6,864 (15)	1,768 (15)	4,513 (17)
70-74	24,323 (29)	13,244 (29)	3,219 (26)	7,860 (29)
75-79	13,990 (17)	7,843 (17)	1,937 (16)	4,210 (16)
≥80 years	10,837 (13)	6,243 (14)	1,637 (13)	2,957 (11)
Hemoglobin A_1c_, %				
<8	14,053 (17)	7,216 (16)	1,081 (9)	5,756 (21)
8-10	11,912 (14)	6,507 (14)	972 (8)	4,433 (17)
≥10	4,079 (5)	2,037 (4)	802 (7)	1,240 (5)
Unavailable^c^	54,676 (65)	29,974 (66)	9,310 (77)	15,392 (57)
Mean hemoglobin A1c (±SD), %^d^	7.95 (1.62)	7.95 (1.57)	8.59 (2.14)	7.79 (1.48)
Diabetes Complications Severity Index^e^ score				
0	47,026 (56)	25,946 (57)	6,384 (52)	14,696 (55)
1	15,783 (19)	8,294 (18)	2,268 (19)	5,221 (19)
2	10,999 (13)	5,892 (13)	1,615 (13)	3,492 (13)
≥3	10,912 (13)	5,602 (12)	1,898 (16)	3,412 (13)
Prescriber Specialty				
Endocrinology	3,652 (4)	1,362 (3)	738 (6)	1,552 (6)
Family practice	33,681 (40)	18,641 (41)	4,595 (38)	10,445 (39)
Internal medicine	29,543 (35)	16,467 (36)	3,832 (32)	9,244 (34)
Nurse/PA	6,889 (8)	3,448 (8)	1,141 (9)	2,300 (9)
Other/Missing	10,955 (13)	5,816 (13)	1,859 (15)	3,280 (12)
Health Plan Type				
Health maintenance organization	25,499 (30)	13,585 (30)	4,070 (33)	7,844 (29)
Preferred provider organization	8,232 (10)	4,612 (10)	965 (8)	2,655 (10)
Other	50,989 (60)	27,537 (60)	7,130 (59)	16,322 (61)
Fill Year				
2013	10,112 (12)	6,033 (13)	1,289 (11)	2,790 (10)
2014	11,766 (14)	6,585 (14)	1,653 (14)	3,528 (13)
2015	11,046 (13)	6,038 (13)	1,379 (11)	3,629 (14)
2016	27,639 (33)	14,907 (33)	4,537 (37)	8,195 (31)
2017	24,157 (28)	12,171 (27)	3,307 (27)	8,679 (32)

BI: basal insulin, SFU: sulfonylurea

^a^ All participant characteristics were significantly associated with initial second-line antidiabetic medication class with a P-value of <0.001

^b^ The Other category comprises DPP-4 inhibitors, GLP-1 agonists, SGLT-2 inhibitors, and thiazolidinediones (TZDs)

^c^ Laboratory values are not routinely available in health plan administrative data sources unless submitted by the laboratory vendor as part of their contract with the health payer. For these data, 35% of submitted laboratory claims nationally included a result. The Unavailable category includes patients without evidence of a test (no claim), as well as those with evidence of a test but no available result

^d^ Mean hemoglobin A1c (± SD) was calculated for all participants with available values

^e^ The Diabetes Complications Severity Index (DCSI) is a validated index composed of micro- and macrovascular complications of diabetes. In this analysis, we modified the DCSI by removing cardiovascular diagnoses that comprise the primary exposure variable. Scores were categorized as 0, 1, 2, and ≥3

**Table 5:** Poor Health Status and Its Individual Components by Initial Second-Line Antidiabetic Medication Class

Health status variables^**a**^	Total	SFU	BI	Other
	N (%)	N (%)	N (%)	N (%)
Poor health^b^				
No	79,544 (94)	43,199 (94)	10,788 (89)	25,557 (95)
Yes	5,176 (6)	2,535 (6)	1,377 (11)	1,264 (5)
End-stage conditions^c^				
No	82,810 (98)	44,712 (98)	11,733 (96)	26,365 (98)
Yes	1,910 (2)	1,022 (2)	432 (4)	456 (2)
Dementia^d^				
No	82,391 (97)	44,597 (98)	11,579 (95)	26,215 (98)
Yes	2,329 (3)	1,137 (2)	586 (5)	606 (2)
Residence in nursing facility^e^				
No	82,631 (98)	44,860 (98)	11,414 (94)	26,357 (98)
Yes	2,089 (2)	874 (2)	751 (6)	464 (2)

*BI* basal insulin, *SFU* sulfonylurea

^a^ All participant characteristics listed were significantly associated with initial second-line antidiabetic medication class with a *P*-value of <0.001

^b^ Poor health was defined according to the following criteria modified from the 2019 Endocrine Society Guidelines for diabetes management in older adults: (1) end-stage conditions; (2) dementia; or (3) residence in a long-term nursing facility

^c^ End-stage conditions included in the 2019 Endocrine Society’s definition of poor health were: metastatic cancer, oxygen-dependent lung disease, chronic kidney disease stages IV or V, end-stage kidney disease, dialysis status, and advanced heart failure. Diagnostic codes used to define these conditions are listed in Table 2

^d^ Diagnosis codes used to define dementia are listed in Table 2

^e^ Residence in a long-term nursing facility was defined using Place of Service (POS) and CPT codes listed in Table 2

## Results

After excluding those with type 1 diabetes (n=28,014), secondary diabetes (n=1,230), and patients who started more than 1 ADM on the index date (n=26,421), the final study cohort included 84,720 adults aged ≥65 years with Medicare Advantage insurance plans. Among all participants with available values, the mean HbA1c prior to starting the index ADM was 7.95% (± 1.62). Over half of participants (54%) received SFU as initial second-line ADM, 14% received BI, and 32% received another ADM. Table 4 reports statistically significant associations between all participant characteristics and index ADM class. Women accounted for a proportionately greater share of BI fills, and men were more likely to fill SFU. Participants aged 80 years and older were more likely to receive SFU and BI than other second-line ADM alternatives. A smaller proportion of SFU users had high DCSI scores and HbA_1c_ values ≥10%, relative to other ADM. Conversely, BI fills were more common among those with a most recent HbA_1c_ ≥10% or unavailable, and higher DCSI score. Most second-line ADM were prescribed by family practice or internal medicine clinicians, who demonstrated proportionally greater prescribing of SFU than other ADM alternatives. Endocrinologists accounted for a greater share of BI prescriptions than SFU. ADM fills were similar according to health plan, with proportionately more ADM alternatives filled in 2017 relative to SFU and BI.

Table 5 displays the bivariate association of the poor health status composite and its individual components with second-line ADM class. Overall, a small proportion of the older adult cohort met the definition of poor health (6%), with a relatively equal share of participants meeting criteria for each of the following individual components that comprised poor health: end-stage chronic conditions (2%), dementia (3%), and residence in a nursing facility (2%). Among those who filled BI as initial second-line ADM therapy, a greater share met the definition of poor health (11%) and each of its individual components, namely end-stage chronic conditions (4%), dementia (5%), and residence in a nursing facility (6%).

In multivariable models (Table 6), the odds of filling SFU or BI were higher for participants in poor health than those in good or intermediate health [OR 1.13 (95% CI 1.05-1.21) and OR 2.34 (95% CI 2.14-2.55), respectively]. Filling SFU as initial second-line ADM therapy was more common among men and in older age categories. By contrast, BI fills were more likely in women and less likely with increasing age categories, except for the oldest age category, which was not significant. SFU and BI were more commonly filled among older adults with poor glycemic control, which followed a ‘dose-response’ relationship with baseline HbA1c levels. This finding was most striking for BI among those with HbA_1c_ ≥10.0% [OR 3.49 (95% CI 3.12-3.90)]. Similarly, BI fills were also associated with diabetes severity as measured by the DCSI, with increasing odds for each successive score. SFU was more likely to be prescribed by family practice and internal medicine clinicians, whereas BI was more commonly prescribed by endocrinologists [OR 1.14 (95% CI 1.03-1.25)]. Greater odds of filling BI was associated with health maintenance organization health plans. Generally, we observed decreasing odds of BI and SFU fills throughout the study period, which was significant for both ADM classes in 2017 compared with 2013.

In a sensitivity analysis removing race/ethnicity from the primary multivariable models, the primary findings did not substantively change. Separate multivariable models among only patients with available HbA_1c_ values, and adjusting for the most recent result, also yielded primary findings that were similar to those reported in Table 6 (data not shown).

**Table 6:** Odds of Receiving SFU or BI as Initial Second-Line Antidiabetic Medication Class by Covariates

Covariate	SFU	BI
	**OR (95% CI)**	**OR (95% CI)**
Poor Health^a^		
No	REF	REF
Yes	1.13 (1.05 - 1.21)	2.34 (2.14 - 2.55)
Sex		
Male	REF	REF
Female	0.88 (0.86 - 0.91)	1.09 (1.04 - 1.14)
Age		
65-67 years	REF	REF
68-70 years	0.98 (0.93 - 1.02)	0.83 (0.77 - 0.89)
70-74 years	1.07 (1.03 - 1.12)	0.85 (0.80 - 0.90)
75-79 years	1.18 (1.12 - 1.24)	0.93 (0.86 - 0.99)
≥80 years	1.32 (1.25 - 1.4)	0.97 (0.89 - 1.04)
Hemoglobin A_1c_^b^		
<8%	REF	REF
8-10%	1.18 (1.12 - 1.24)	1.19 (1.08 - 1.31)
≥10%	1.33 (1.23 - 1.44)	3.49 (3.12 - 3.90)
Unavailable	1.53 (1.47 - 1.60)	3.35 (3.12 - 3.60)
Diabetes Complications Severity Index^c^		
0	REF	REF
1	0.92 (0.89 - 0.96)	1.09 (1.02 - 1.15)
2	0.95 (0.91 - 1.00)	1.12 (1.04 - 1.20)
≥3	0.92 (0.87 - 0.96)	1.25 (1.16 - 1.34)
Prescriber specialty		
Family practice	REF	REF
Endocrine	0.50 (0.47 - 0.54)	1.14 (1.03 - 1.25)
General/Internal	1.00 (0.96 - 1.03)	0.95 (0.90 - 1.00)
Nurse/PA	0.85 (0.80 - 0.90)	1.10 (1.02 - 1.20)
Other/Missing	0.99 (0.95 - 1.04)	1.21 (1.13 - 1.30)
Health Plan Type		
Other	REF	REF
Health maintenance organization	1.03 (0.99 - 1.07)	1.25 (1.18 - 1.31)
Preferred provider organization	1.06 (1.00 - 1.11)	0.88 (0.81 - 0.96)
Fill Year		
2013	REF	REF
2014	0.86 (0.81 - 0.92)	1.08 (0.98-1.18)
2015	0.80 (0.75 - 0.85)	0.90 (0.82 - 0.99)
2016	0.90 (0.85 - 0.95)	1.28 (1.18 - 1.39)
2017	0.69 (0.65 - 0.73)	0.91 (0.84 - 0.99)

*BI *basal insulin, *SFU *sulfonylurea

^a^ Poor health was defined according to the following criteria modified from the 2019 Endocrine Society Guidelines for diabetes management in older adults: (1) end-stage conditions; (2) dementia; or (3) residence in a long-term nursing facility

^b^ Laboratory values are not routinely available in health plan administrative data sources unless submitted by the laboratory vendor as part of their contract with the health payer. For these data, 35% of submitted laboratory claims nationally included a result. The Unavailable category includes patients without evidence of a test (no claim), as well as those with evidence of a test but no available result

^c^ The Diabetes Complications Severity Index (DCSI) is a validated index composed of micro- and macrovascular complications of diabetes. In this analysis, we modified the DCSI by removing cardiovascular diagnoses that comprise the primary exposure variable. Scores were categorized as 0, 1, 2, and ≥3

## Discussion

Practice guidelines for managing diabetes in older adults increasingly emphasize an individualized approach for determining appropriate glycemic targets and antidiabetic medication (ADM) regimens [3–7]. When making treatment decisions, clinical guidelines universally recommend assessing older patients’ health status and considering potential associated risks, such as hypoglycemia [3–7]. Our study of 84,720 older adults found that the odds of filling SFU or BI was higher for participants in poor health than those in good or intermediate health, even after adjustment for the most recent HbA_1c_. Filling these same ADM classes was also more common among other subgroups of older adults. For example, SFU fills increased with advancing age; and BI fills increased with greater numbers of diabetes complications. Collectively, these findings demonstrate that SFU and BI remain widely used in older adults, including those who are particularly vulnerable for experiencing adverse events.

This analysis offers a unique perspective into real-world prescribing patterns for second-line ADM in older adults, with findings that are similar to a recent national analysis of electronic medical records [23]. Our use of administrative claims data enabled capture of medication fills, rather than prescription orders that are available from electronic health records but provide limited evidence about whether patients fill the medication. There is little research investigating the gap between existing practice patterns and current consensus guidelines on ADM use in older adults.

The poor health group in our analysis is of particular interest and has received little prior study, given that this population is almost universally excluded from clinical trials. Our findings suggest that SFU and BI are used frequently among older adults in poor health, which conflicts with multiple clinical guidelines recommending less frequent use of these medications among vulnerable older adults. One reason for this finding may be related to these medications’ potent glucose-lowering effects [19]. However, short-term adverse events, including hypoglycemia, are frequently described among older adults in poor health, thereby compromising the future benefit of glycemic control in this group. Providers must consider the time horizon for treatment benefit in achieving glycemic targets and minimizing microvascular and macrovascular complications, such that therapy goals are attainable within patients’ life expectancy, and treatment benefit outweighs risk of harm. Based on this assessment of risks and benefits, treatment with SFU and BI may still be appropriate among some older adults in poor health.

Previous studies have demonstrated that older adults with diabetes often receive more aggressive treatment than is warranted, reaching HbA_1c_ values below recommended targets [11, 13, 16, 17, 24]. Further, prior studies show low rates of medication de-intensification in older adults, highlighting a missed opportunity to reduce over-treatment [14, 15]. Several studies evaluate diabetes treatment according to older adults’ health status. However, prior research has not described medication fills using the consensus framework that categorizes patients based on end-stage comorbid conditions, dementia, and/or residence in long term care [11]. Because providers are accustomed to evaluating these comorbidities and characteristics [18, 25], the diabetes treatment framework originally developed by Blaum et al. [18] may prove useful in guiding development of individualized ADM regimens for older adults. Adherence to this framework, and resulting patient outcomes, should be studied empirically in future research that evaluates its implementation. Our findings demonstrate that, despite consensus guidelines from the AGS, ADA, and Endocrine Society recommending use of this framework, older adults in poor health were often treated with SFU and BI, highlighting the persistent gap that this recommendation aims to address.

This paradox is complex, and understanding it more completely requires further study. However, a few explanations and trends are evident. The enduring and frequent use of SFU therapy in older adults may be related to several factors, including its longstanding availability, prescriber familiarity, patient preference, significant glucose lowering effects, and low cost compared to many newer ADM alternatives [19]. The latter may be an especially important driver of SFU use, as these medications have the lowest cost of all ADM classes studied here [19]. Basal insulin is also an established therapy that may be an appropriate patient-centered treatment, especially for those with significantly elevated HbA_1c_ or contraindications to alternative therapy, such as end stage renal disease [19]. Prior research found that older adults with CKD, heart failure, and cardiovascular disease are less likely to fill SGLT-2 medications than those of younger age and those without such comorbid conditions, despite well documented clinical benefits [26]. It is not known whether this is related to concerns about side effects from newer medication classes among patients or providers, their higher cost, or providers’ greater familiarity and experience with older ADM classes. Our study may have shown lower use of SGLT-2 and GLP-1 because the study period ended shortly after the publication of seminal trials demonstrating their cardiovascular and renal benefits. Using current data, future research must evaluate other reasons for infrequent use of newer ADM classes among older adults, and study ways to increase their uptake when clinically indicated.

Our study has notable limitations. Observational research is prone to confounding, which may limit causal inference from the findings. However, this issue is of greater potential concern when examining clinical outcomes rather than prescribing patterns examined in the current study. Our claims data source did not enable assessment of whether SFU and BI was clinically appropriate for some participants. Electronic health data have the potential to answer questions about the appropriateness of treatment decisions, given more available clinical information than claims. Hypoglycemia or falls were not examined as clinical outcomes here because these are underreported in claims data, and our group recently published an analysis focusing on glycemic outcomes of ADM using the same data source [10, 27]. Claims data do not routinely include laboratory results, although we were able to examine lab data for approximately one-third of participants. While we were unable to fully explore the relationship between HbA_1c_ level and choice of second-line ADM, the primary findings were unchanged after adjusting for the most recent HbA_1c_ result among those with available HbA_1c_ data.

We analyzed data on participants’ health plans, but did not have information about formularies or out-of-pocket costs that are known to influence ADM treatment decisions [28]. We used widely accepted definitions for dementia using diagnosis codes outlined by the Centers for Medicare and Medicaid Services [29]. These definitions may not have captured all participants with this exposure in our study, thereby underestimating the number of older adults in poor health. However, prior research on Medicare beneficiaries found that diagnosis codes are more sensitive for identifying older adults with dementia, which comprises the poor health group in the current study, than those with mild cognitive impairment [30]. Detailed data on functional impairment, which is another component of poor health status, is not available in administrative claims data. Prior studies have found that using diagnosis codes to define functional impairment exhibits poor sensitivity and specificity [31]. Clinical evaluation of functional status includes performance-based testing or survey data, which should be included in future research to capture patients with this exposure most completely.

## Conclusions

In conclusion, our study documents high rates of SFU and BI use among all older adults and among particularly vulnerable subgroups, including those with advanced age, dementia, and high rates of diabetic complications. Our findings demonstrate a large opportunity to close the gap between real-world ADM prescribing patterns and current consensus guidelines that emphasize less frequent use of SFU and BI in older adults. This is especially true for those in poor health, for whom providers should consider alternative therapies and strategies for de-intensifying treatment. When initiating second-line ADM therapy, clinicians should follow recommended approaches for patient-centered care, using shared decision-making to discuss potential risks and benefits of different treatment options [32]. Future research should examine whether ADM prescribing patterns change in reaction to recent clinical guidelines. These studies should explore alternate data sources that allow for more complete capture of factors that define poor health in older adults. Future research on this topic will be informed by data from an ongoing clinical trial comparing the glycemic effects of most ADM classes studied here [33]. Population health efforts have the potential to improve clinical practice by examining data across health systems and using clinical decision support or other interventions that promote appropriate prescribing of SFU and BI. Emerging policies focused on reducing hypoglycemia also offer opportunities to align with diabetes treatment guidelines and incentivize care that limits adverse medication outcomes among older adults.

## Data Availability

The datasets generated during and/or analyzed during the current study are available from the corresponding author on reasonable request.

## Abbreviations

ADM: Antidiabetic medications
SFU: Sulfonylureas
BI: Basal insulin
ADA: American Diabetes Association
AGS: American Geriatrics Society
DPP-4: dipeptidyl peptidase 4
GLP-1: glucagon-like peptide-1 angonists
SGLT-2: Sodium-glucose cotransporter 2 inhibiors
TZD: Thiazolidinediones
HbA_1c_: Hemoglobin A_1c_
STROBE: Strengthening the Reporting of Observational Studies in Epidemiology
DCSI: Diabetes Complications Severity Index
